# The A. M. A. Meeting in Denver

**Published:** 1898-08

**Authors:** 


The A. M. A. Meeting in Denver.—The recent meeting of the American Medical Association in Denver was a great success, in the matter of attendance, the amount of scientific work performed, and the strengthening of the bonds of brotherhood among the members. The general sessions were presided over by Dr. T. J. Happel, of Tennessee, and the business of these meetings was dispatched in a way very satisfactory to the members.

Denver is very fortunately situated for the entertainment of a meeting of this kind, and the citizens, and especially the profession of Denver, deserve great praise for the very royal manner in which they entertained the delegates and their families. There were a number of banquets and receptions at the Brown Palace Hotel, and at the private residences of several of the leading citizens, including Mr. and Mrs. Kountze, Judge and Mrs. E. P. Hill, Mr. and Mrs. Campion, and Dr. and Mrs. Hershey. All were most cordially received, and so far as the entertainers were concerned, nothing was left undone to make each caller's enjoyment full and complete. The one feature which marred the pleasure of these entertainments was the duplicity and rascality of the Denver hackmen, or rather the owners of carriages. The complaints on this score were many and loud. The profession of Denver, however, were in no way responsible for these acts of robbery, and that they exerted themselves to the utmost to entertain their guests is amply attested by the many entertainments furnished, including a trip up Clear Creek Canon, over "The Loop," to Silver Plume, also an excursion to Colorado Springs and Manitou. These excursions were well patronized by the delegates and their wives, and all were wonderfully delighted with the magnificent scenery and the royal hospitality of the entertainers. On the "loop" trip the citizens of Idaho Springs furnished an elegent luncheon to the many hundreds of excursionists.

After the adjourment of the association many of the members and visitors availed themselves of the cheap excursion rates to Cripple Creek, Glenwood Springs and Salt Lake City. The liberality of the various railroad companies, especially the Denver & Rio Grande, the Colorado Midland and Rio Grande Western, in furnishing low excursion rates and the best of accommodations and service meet with the hearty appreciation of the excursionists.

This meeting will be long remembered by those who attended it. The hospitality of the people, the abundant intertainments, the magnificent scenery, and the delightful climate of Colorado made a lasting impression on the visitors and one and all have gone away with the most pleasant recollections of Colorado and her citizens.

The Association will meet next year in Columbus, Ohio.

The newly elected officers of the American Medical Association are: President, J. M. Mathews, Louisville; First Vice President, W. W. Keen, Philladelphia; Second Vice-President, J. W. Graham, Denver; Third Vice-President, H. A. West, Galveston; Fourth Vice-President, J. E. Minney, Topeka; Secretary, W. B. Atkinson, Philadelphia; Treasurer, H. P. Newman, Chicago; Librarian, G. B. Webster, Chicago; Board of Trustees, Alonzo Garcelon, I. N. Love, H. L. E. Johnson, T.

J. Happel; Judicial Council, S. Bailey, D. R. Brower, N. S. Davis, H. D. Didama, D. Mason, F. T. Rogers, M. B. Ward, W. B. Jones. Dr. J. C. Wilson of Philadelphia, has the general address on Medicine; Dr. Floyd McCrea, of Atlanta, the address on surgery; Dr. D. R. Brower, of Chicago, that on state medicine.

---

OUR HOUSTON CONTEMPORARY says that Dr. Austin Flint, Sr., has resigned from Bellevue Hospital Medical College. Dr. Austin Flint, Sr., died several years ago. Dr. Austin Flint, Jr., the *physiologist*, was doubtless meant. Dr. Red ought to be better posted, especially as he has recently spent six weeks at Philadelphia, in medical circles. Such is fame.

